# A Genome-Wide Association Study Identifying Single-Nucleotide Polymorphisms for Iron and Zinc Biofortification in a Worldwide Barley Collection

**DOI:** 10.3390/plants11101349

**Published:** 2022-05-19

**Authors:** Solange Nyiraguhirwa, Zahra Grana, Hassan Ouabbou, Driss Iraqi, Mohammed Ibriz, Sujan Mamidi, Sripada M. Udupa

**Affiliations:** 1 International Center for Agriculture Research in Dry Areas (ICARDA), Rue Hafiane Chekaoui, P.O. Box 6299, Rabat 10000, Morocco; hirwaso@gmail.com (S.N.); zahra.g02@gmail.com (Z.G.); 2 Institut National de Recherche Agronomique (INRA), Avenue Ennasr, P.O. Box 415, Rabat 10080, Morocco; hassan.ouabbou@gmail.com (H.O.); iraqid@yahoo.fr (D.I.); 3 Faculty of Sciences, Ibn Tofail University, University Campus, P.O. Box 133, Kénitra 14000, Morocco; m_ibriz@yahoo.fr; 4 Hudson Alpha Institute for Biotechnology, 601 Genome Way Northwest, Huntsville, AL 35806, USA; sujan.mamidi@gmail.com

**Keywords:** association mapping, barley, biofortification, *Hordeum vulgare* L., GWAS, iron, micronutrients, SNP, zinc

## Abstract

Micronutrient deficiency affects half of the world’s population, mostly in developing countries. Severe health issues such as anemia and inadequate growth in children below five years of age and pregnant women have been linked to mineral deficiencies (mostly zinc and iron). Improving the mineral content in staple crops, also known as mineral biofortification, remains the best approach to address mineral malnutrition. Barley is a staple crop in some parts of the world and is a healthy choice since it contains β-glucan, a high dietary protein. Barley mineral biofortification, especially with zinc and iron, can be beneficial since barley easily adapts to marginalized areas and requires less input than other frequently consumed cereals. In this study, we analyzed zinc and iron content in 496 barley samples. The samples were genotyped with an Illumina 50 K SNP chip. Genome-wide association studies (GWAS) identified 62 SNPs and 68 SNPs (*p* < 0.001) associated with iron and zinc content in grains, respectively. After a Bonferroni correction (*p* < 0.005), there were 12 SNPs (single-nucleotide polymorphism) associated with Zn and 6 for iron. SNP annotations revealed proteins involved in membrane transport, Zn and Fe binding, linked to nutrient remobilization in grains. These results can be used to develop biofortified barley via marker-assisted selection (MAS), which could alleviate mineral malnutrition.

## 1. Introduction

Agriculture production for staple crops has increased since the green revolution, which prevented hunger for millions of people worldwide. However, these staple crops lack essential micronutrients such as iron, vitamin A, vitamin D, zinc, and iodine. As a result, a lack of meal diversity is the leading cause of malnutrition, termed “hidden hunger”. Malnutrition remains a worldwide crisis, but it is especially severe in developing countries, with the most damaging nutritional deficiencies being iron (Fe) and zinc (Zn). A third of the population worldwide suffers from zinc deficiency, while more than half lack iron [1]. Deficiencies in vitamins and minerals cause severe health problems such as weak immunity and poor growth, mainly in children under five years old and pregnant women. The WHO [2] estimates that 45% of children’s deaths can be attributed to malnutrition. Anemia, caused primarily by iron deficiency, affects about 43% of children worldwide [3].

Ways to alleviate malnutrition include the biofortification of crops, increased meal diversity, fortification of products, and the use of supplements. Biofortification of staple crops is the most sustainable and relatively low-cost way to alleviate malnutrition since poor farmers in rural areas can benefit from eating their crops. However, meal diversity, supplements, and fortification are expensive, hard to deliver, and unsustainable. With this growing importance for biofortification, Harvestplus, a Consultative Group on International Agricultural Research (CGIAR) Research Program, works on increasing micronutrient content such as zinc, iron, and provitamin A in crops that are important for developing countries [4].

Barley (*Hordeum vulgare*) is a staple food in some parts of the world. Its consumption ranges between 2 and 36 kg/person in North Africa, Ethiopia, and central and East Asia [5]. In addition, barley has other important uses, including as livestock feed and malt in the brewing industry. Its grain contains 65 to 68% starch, 10 to 17% proteins, 2 to 3% fat, 4 to 9% β-glucan, vitamins, and about 1.5 to 2.5% minerals. The uptake of barley as human food has increased recently due to its health benefits. It is rich in β-glucan, a dietary fiber. It lowers the risk of cardiovascular diseases, increases satiety, reduces cholesterol, promotes weight loss, and regulates insulin [6]. Barley food products also supply essential micronutrients needed for humans, such as Fe and Zn. However, their content in barley grains and other staple crops is often low. For this reason, improving zinc and iron in grains through breeding is essential to tackle micronutrient malnutrition. Barley remains a valuable crop for biofortification since it tolerates various stresses and climate conditions, requires less input than other cereals and offers several health benefits [7,8].

The objective of breeding biofortified cereals is to improve micronutrient content in grains and increase their bioavailability by breeding for the phytase enzyme that reduces anti-nutrients. Breeding for micronutrients begins by accessing micronutrient variation, selecting high micronutrient content varieties, and deploying them in a breeding program. Fe and Zn levels in barley grains are generally higher in wild barley, followed by landraces and then cultivars. Landraces and wild barley harbor many important alleles that can be used to improve micronutrients [8]. Our study aims to identify the variation of zinc and iron in 496 barley germplasms. We use the GWAS approach to identify SNP associated with iron and zinc content, which can be used to develop improved cultivars rich in zinc and iron. In addition, this study identifies candidate genes that can be used for functional studies.

## 2. Results

### 2.1. Phenotype Results

**Figure 1:**
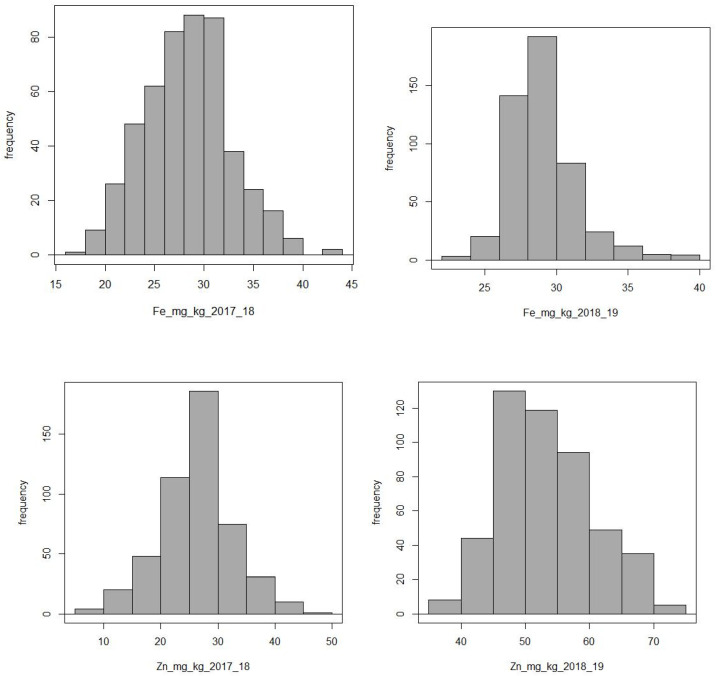
Distribution of iron (Fe) and zinc (Zn) in barley grains for two crop seasons.

Our barley collection showed a wide range of iron and zinc content in grains (Figure 1). The range of Fe is 16.75 mg/kg to 43.15 mg/kg, with a mean of 28.75 mg/kg. On the other hand, zinc had a range of 6.28 mg/kg to 70.911 mg/kg with a mean of 39.58 mg/kg. Fe and Zn content for both crop seasons and their descriptive statistics are in supplementary Table S1. The frequency distributions with repeated checks, box plots, and descriptive statistics of adjusted means for zinc and iron are presented in supplementary figures (Figures S2–S5). High Zn was observed in the 2018–2019 crop season grown at the ICARDA research station, Marchouch, near Rabat, Morocco.

### 2.2. Population Structure and Linkage Disequilibrium

**Figure 2:**
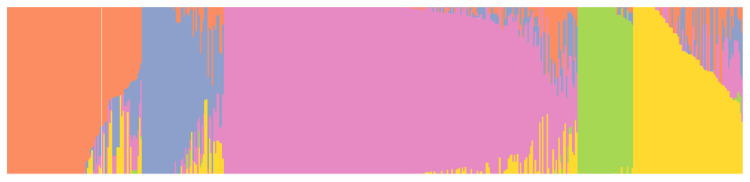
Five subpopulations revealed by fastStructure software resulting from 14,015 SNP markers and 496 barley germplasms.

**Figure 3:**
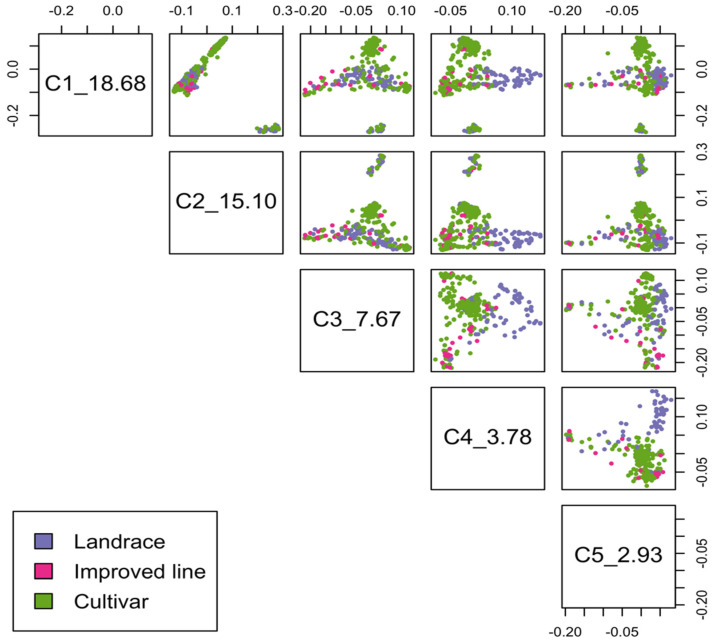
MDS plot by population type for 495 barley samples with 14,015 SNP markers in plink1.9 software.

Five subpopulations were identified, and the samples were not grouped by geography or by the type of genetic material (cultivar or landrace) (Figure 2). Subpopulation 3 had the highest number of samples among other subpopulations, and they were primarily landraces. Subpopulation 5 had more cultivars (52) than the other subpopulations (Table 1). However, multidimensional scaling (MDS) plots by population type showed the separation of landraces from cultivars and improved lines in second to later components. The cultivars and improved lines clustered together (Figure 3). The first five components explained 48% of the cumulative variation. They did not split populations into discrete groups, suggesting that the population structure was not strong and GWAS was feasible.

For an r^2^ equal to 0.2, the average Linkage disequilibrium (LD) decay was about 200 Kbp (Supplementary Figure S1). This distance is used to define the window size for searching for candidate genes in the GWAS analyses.

### 2.3. GWAS Results

**Figure 4:**
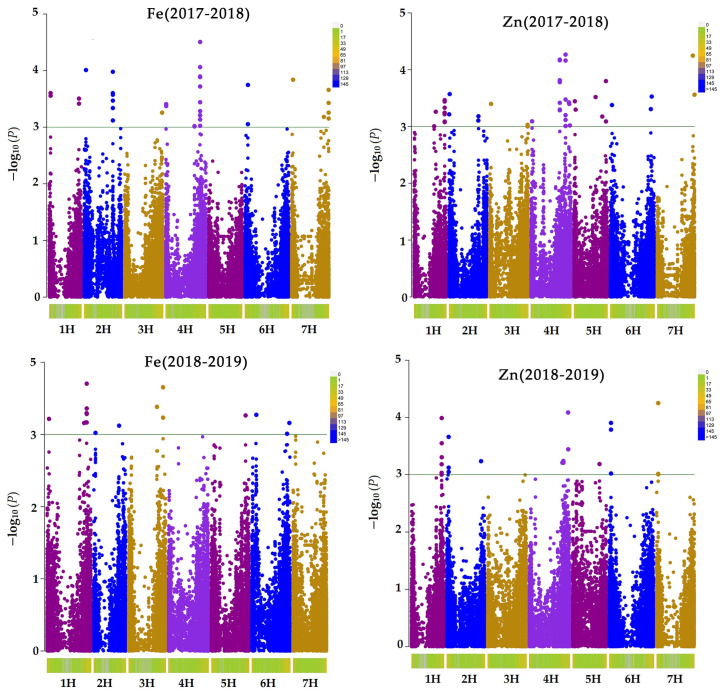
The Manhattan plot reveals significant SNPs (*p* < 0.001) associated with Fe and Zn content. The (−*log*_10_ *p*) values on the Y-axis are plotted against the chromosome position on the X-axis. The threshold is the blue horizontal line set at −*log*_10_ *p* = 3.

**Figure 5:**
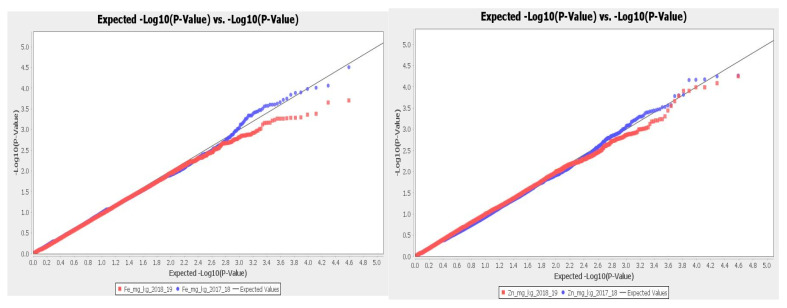
Quantile-quantile (Q-Q) plots for Fe (**left**) and Zn (**right**).

This study identified 62 and 68 significant SNPs (*p* < 0.001) associated with iron and zinc, respectively. At (*p* < 0.005) after Bonferroni correction, the number of SNPs were 12 and 6 for Fe and Zn, respectively. Table 2 and Table 3 display their phenotypic variation (R^2^), *p*-value, position, the adjusted *p*-value after the Bonferroni correction, and other associated information such as the candidate gene. The quantile-quantile (Q–Q) plot and Manhattan plot for both Zn and Fe are displayed in Figure 4 and Figure 5.

Annotations of significant SNPs after Bonferroni correction (*p* < 0.005) using BARLEX and BarleyVarDB are displayed in Table 2 and Table 3. They correspond to candidate genes such as Tetratricopeptide repeat protein 7A, involved in metal (zinc) binding [9]. Nascent polypeptide-associated complex subunit alpha-like protein 3, which plays a key role under zinc stress, has been related to zinc stress in *Arabidopsis thaliana*. Nascent polypeptide-associated is also expressed under Fe deficiency [10,11].

All significant SNPs (*p* < 0.001) are displayed in supplementary Tables S2 and S3 with their chromosomal location, position, and *p*-value. They are involved in several functions related to metal transport, ion binding, and remobilization of Fe and Zn into grains. For example, candidate genes such as the cytochrome P450 superfamily participate in element transport [12]. Other proteins expressed in low Fe and Zn conditions, including acyl-ACP thioesterase and ribosomal protein, respectively, were identified in this study, [13,14] (Supplement Tables S2 and S3).

## 3. Discussion

Nutrition is essential for good health, overall well-being, quality of life, and overall work productivity. However, around two billion people worldwide lack access to nutrient-rich foods. Nutritious essential compounds that are often missing or inadequate in staple crops include microelements, vitamins, unsaturated fatty acids, and essential amino acids. Deficiency in minerals is mainly observed in Zn and Fe, making them suitable for mineral biofortification [3]. Several genes are involved in Fe and Zn uptake, transport, and accumulation in the edible parts of the plants, e.g., grains. Therefore, it is essential to understand the mechanism and identify genetic loci associated with micronutrient content in seeds. GWAS is a widely used approach to identify loci and new alleles associated with phenotypic traits.

This study investigated the zinc and iron content in grains of 496 barley accessions, predominantly consisting of landraces, which can be directly used in breeding programs to introgress the necessary loci. We found that iron ranged between 16.75 and 43.15 mg/kg, and zinc concentration varied between 6.28 mg/kg and 70.911 mg/kg. Zinc and iron content in barley grains varied for both locations. The variation observed in iron and zinc concentrations may be explained by moisture content, soil type, and soil treatment differences between the two crop seasons and locations [15].

Gyawali et al. [16] analyzed the content of iron, zinc, and other elements in 336 improved barley lines. They showed that the zinc content in grains ranged from 10.4 to 54.5 mg/kg, which is lower than our collection. In their collection, the Fe content in grains varied from 21.9 to 91.0 mg/kg, which is more than our collection. Mamo et al. [17] analyzed the Fe and Zn content in the Eritrean and Ethiopian barley landraces. They found that the iron content ranged from 27.26 to 109.60 mg/kg and the zinc content ranged between 19.69 and 87.42 mg/kg, which is much higher than we observed in our study. Their high Fe and Zn contents can be explained by the fact that Eritrean and Ethiopian barley landraces are known to be rich in micronutrients. Landraces, in general, have been proven to be higher in micronutrient content. For instance, Moroccan landraces were found to be high in zinc and iron [18]. Several other studies found higher concentrations of micronutrients in wild barley than in cultivated barley. For instance, Ref. [19] found a range of 66.30 to 493.90 mg/kg of Zn and 10.80 to 329.10 mg/kg of iron in wild barley grains. In a subset of the wild barley nested association mapping (NAM) population, the iron content in grains ranged between 29.9 mg/kg and 35.2 mg/kg. In contrast, the zinc content was between 19.8 and 26.7 mg/kg [20]. Wild barley can then be exploited for desired traits, but their crossing with elite lines brings undesirable alleles.

The SNP annotation revealed the identification of candidate genes involved in Fe and Zn transport and their accumulation in grains. Proteins such as nascent polypeptide-associated complex subunit alpha-like protein 3 are expressed in low Fe barley [9]. On the other hand, we have identified important candidate genes for SNPs (*p* < 0.001). They correspond to genes such as the cytochrome P450 superfamily protein, which is involved in element transport, iron and zinc binding, and is found in genotypes with a higher Fe and Zn content [12]. This study identified serine/threonine-protein kinase, which is an ATP and protein binder [21]. Acyl-ACP thioesterase is expressed in low Fe [13]. Receptor-like protein kinase 4 is induced in heavy metal stress [22]; the NAC domain protein is linked to nutrient remobilization in grains [23] (see Table 2 and Table 3 and Supplementary Tables S2 and S3 for the complete list of annotated genes).

We identified 68 SNPs (*p* < 0.001) associated with Zn on all seven barley chromosomes. After the Bonferroni correction to reduce false SNPs, there were 12 remaining SNPs. Lonergan et al. [24] found QTL on chromosomes 1HS, 2HL, and 5HL was associated with zinc content in grain from the DH population. Our study confirmed their finding; we found SNP SCRI_RS_182603 at 15065044 bp (27.27 cM) on 1H, 4 SNP at655 Mb −656 Mbp (81cM) on 2H, and SNP JHI-Hv50k-2016-298724 at 348 Mbp (44 cM) on 5H. Sadeghzadeh et al. [25] identified 2 QTL associated with Zn content in the barley DH population grains from field experiments. Their studies mapped QTLs associated with Zn on 2HS at 23.7 cM. Our study also mapped 3SNP associated with Zn at 31 Mbp (23.16 cM) in this region on chromosome 2H. In the same region on chromosome 2H, [26] and [27] found a zinc transporter gene 8 (HORVU2Hr1G025400) at 725,227,365 bp associated with high Zn, a homolog of the *Arabidopsis thaliana* ZIP1gene and rice OsZIP3 gene. Both genes are members of the family of zinc-regulated transporters and iron-regulated transporter protein (ZIP), which are known to contribute to Zn uptake [28]. The closest marker to this region in our study is the SNP JHI-Hv50k-2016-127169, located at 721,667,630 bp. Detterbeck et al. [27] also identified a QTL on chromosome 2H at 82.8 cM. Two yellow stripe-like (YSL) transport genes were annotated on this chromosome at 80.89 cM and 80.95 cM for MLOC_40066.1 and MLOC_61170.4, respectively. They have been associated with Zn and Fe transport and other elements that adhere to nicotianamine transport and phytosiderase [29]. We found four SNP markers, three at 81.52 cM and one at 81.8 cM (655 Mbp and 656 Mbp), close to the position of these YSL genes. In addition, Mamo et al. [17] identified four SNPs on chromosome 6H associated with Zn content at 122 cM and 128 cM. We found an SNP associated with Zn in 6HL near that region.

Gyawali et al. [16] identified 46 QTLs associated with multiple elements in barley grains using 336 diverse improved lines of the ICARDA barley breeding program. A total of 11 QTLs for Fe and 3 QTLs for Zn content in grains were identified among them. On chromosome 5H, [16] the identified QTL E-5H-44.99, associated with Zn, was found at 44.99 cM. In addition, we found an SNP JHI-Hv50k-2016-298724 associated with Zn at 44.17 cM in this region on chromosome 6H. 

Our study identified 62 SNPs associated with Fe on all barley chromosomes except 6H (*p* < 0.001). After the Bonferroni correction (*p* < 0.005), 6 SNPs remained associated with Zn. On chromosome 3H, we found 8 SNPs at 595 Mbp (83 cM), which are in the region of the QTL Fe-3H-83.63 identified earlier by [16]. Moreover, we found 2 SNPs at 514 Mbp close to the QTL (Fe-1H-90.04, Fe-1H-87.87) identified by [16] on chromosome 1H associated with Fe in grains. In another study, 15 elements in barley grains, including Zn and Fe, were analyzed using association mapping in a wild barley NAM population and identified several QTLs. These QTLs were detected on all seven barley chromosomes for both Fe and Zn [30].

Besides conventional breeding for micronutrients, barley biofortification in Zn and Fe can be achieved or complemented with agronomic approaches, such as applying soil and leaf fertilizers, adjusting soil pH, rotating crops, and using microorganisms that fix nitrogen or solubilize Zn (e.g., Bacillus licheniformis) [31]. However, agronomic approaches require recurrent expenses. Genetic transformation has shown an increase in micronutrients. For instance, overexpressing a Zn protein transporter (ZIP) transgenic barley showed a higher zinc content in the barley in soil high in Zn [32]. However, transgenic crop acceptance and regulation remain a challenge—for instance, the acceptance of golden rice developed by genetic transformation.

The bioavailability of Zn and Fe can be limited by anti-nutrients such as phytic acid or phytate. Breeding for their low quantity in crops and breeding for phytase enzymes that degrade phytic acid will improve the bioavailability of Fe and Zn [33]. Mature grain phytase activity was evaluated using the genome-editing tool. Clustered regularly interspaced short palindromic repeats (CRISPR)/Cas9 combined with transcription activator-like effector nucleases TALENs in barley by mutating the phytase gene promoter [34].

Barley resists Fe deficiency more than other cereals; the genes involved can be adapted to other cereals. For instance, nicotianamine aminotransferase genes (NAAT genes) intervene in the liberation of phytosiderophores (e.g., mugineic acid MA,2-deoxymugineic acid DMA), which are used by the plant to uptake Fe. They are expressed in high quantities in barley when the iron content is low. Takahashi et al. [35] observed tolerance to Fe deficiency in transgenic rice harboring NAAT genes from barley.

## 4. Materials and Methods

### 4.1. Plant Materials and Experimental Design

This study used a collection of 496 spring barley (*Hordeum vulgare* ssp. *Vulgare)* genotypes that included 95 cultivars, 28 improved lines, and 373 landraces. Of those, 419 lines are 6-row, and others are 2-row. Among the 496 lines, 105 are naked, while 6 are black seeds. Seeds originating from different countries were obtained from the USDA, ARS, and Plant Genetic Resources Unit (PGRU) (Supplementary Table S1). The field evaluation for the 2017–2018 and 2018–2019 crop seasons was conducted at the ICARDA Marchouch field research station (latitude 33.607949; longitude −6.705882; altitude 410 m). The experiment design used for both crop seasons was an augmented complete block design with six checks. Seed samples were randomly taken from each plot. Harvesting and threshing were carried out manually to avoid any elemental contamination. For estimating the adjusted mean values for each trait using the phenotype values for the controls, we used the aug.rcb function from the plant breeding package in R (https://plantbreeding.r-forge.r-project.org/ accessed on 1 May 2022).

### 4.2. Iron and Zinc Content

The iron (Fe) and zinc (Zn) content analyses were carried out at the Cereal and Quality Laboratory at ICARDA, Rabat, Morocco. Barley seeds from the 2017–2018 crop season were analyzed with inductively coupled plasma-optical emission spectrometry (ICP-OES; Model iCAP-7000 Duo, ThermoFisher Scientific, Waltham, MA, USA) following the modified HNO_3_ and H_2_O_2_ methods [36,37].

Clean and dried barley seeds obtained from each plot were ground in a clean cyclone sample mill (Twister, 10 mm−250 μm, Retsch, Haan, Nordrhein-Westfalen, Germany). Flour samples (500 mg) were placed in a clean digestion tube, and 8 mL of nitric acid (70% HNO_3_) was added and incubated overnight. The tube was placed in a digestion bloc (QBlock series, Horiba, Kyoto, Japan) for 1 h at 90 °C, then shaken for 15 min and 45 min. Then, we added 3 mL of hydrogen peroxide (H_2_O_2_) to each tube at 90 °C. At the end of digestion, as characterized by the solution’s discoloration and the discontinuation of brown smoke, the tube was removed from the digestion block and cooled at room temperature. The sample was filtered through Whatman papers, diluted at 1:10 with 6M of HCl, and then loaded into the ICP-OES machine to estimate Fe and Zn. Due to available funding, we could not analyze both crop seasons with ICP. For barley seeds of the 2018–2019 crop season, iron and zinc concentrations in barley grain were determined using X-ray fluorescence spectroscopy (X-supreme 8000, Oxford Instruments, Abingdon, UK). The X-ray fluorescence spectroscopy was calibrated using the inductively coupled plasma-optical emission spectroscopy (ICP-OES); (iCAP-7000 Duo, Thermo Fisher Scientific) following a modified HNO_3_-H_2_O_2_ method [38].

### 4.3. Genotyping

DNA was extracted from young barley leaves following the CTAB (cetyltrimethylammonium bromide) protocol [39] with a slight modification [40]. A total of 496 accessions were genotyped with barley 50 K iSelect SNP arrays from Illumina at USDA-ARS, Fargo [41]. After genotyping, these markers were initially filtered for 20% of missing data. The data was then imputed in the fast phase [42] using default settings. These markers were further filtered for a minor allele frequency (MAF) of 0.05 to be used for GWAS. 

### 4.4. Population Structure, Kinship and Linkage Disequilibrium

Population structure (k1-k15) was estimated in fast structure [43] using a random set of 25,000 markers. For the selection of 25,000 markers, we first removed markers that were in LD using plink 1.9 ([44]; https://www.cog-genomics.org/plink/1.9/accessed on 1 May 2022) with the parameters “--indep-pairwise 50 50 0.5”. Then, a random set was selected to estimate population structure using the fast structure and multidimensional scaling (MDS) in plink 1.9. Next, kinship was calculated in TASSEL 5.0 (Trait Analysis by Association, Evolution, and Linkage) using centered IBS [45]. Using TASSEL 5.0, principal components (PC) were also estimated to control for population structure in GWAS.

### 4.5. LD Decay

The extent of linkage disequilibrium (LD) for the population was determined as described in [46]. For this, we first calculated LD (r2) using plink (--ld-window 1000 --ld-window-kb 5000). The r2 value was averaged every 500 bp of distance. A nonlinear model was fit for this data in R, and the extent was determined as to when the LD (r2) nonlinear curve reached 0.2.

### 4.6. Genome-Wide Association Analysis (GWAS) 

GWAS was performed in Tassel 5.0 software [45] using different models. One model was without population structure (PC) and kinship (termed Naive), one other model controlled only for population structure (PC’s), and one other model was used that only considered kinship as a covariate. The GLM (Naive model) model failed to control false positives very well. The MLM model using PC and kinship as covariates showed efficacy in finding true associations and control population structure and was used in this study. The Bonferroni correction model was used to reduce false SNPs (*p* < 0.005). Significant SNPs were annotated with the BARLEX database at https://apex.ipk-gatersleben.de/apex/f?p=284:10 (accessed on 1 May 2022) for candidate genes [47]. The BarleyVarDB database [48] at http://146.118.64.11/BarleyVar/ (accessed on 1 May 2022 and Barley map [49] at http://floresta.eead.csic.es/barleymap/ (accessed on 1 May 2022 were used to find their function.

## 5. Conclusions

This study used a GWAS approach to map genetic loci associated with Zn and Fe content in barley grains. A collection of 496 diverse barley lines was screened for zinc and iron content in grains in 2 locations for 2 crop seasons. The Fe and Zn content in grains showed a wide range of variations. We identified genotypes that showed high Zn and Fe content in grains; they constitute the best lines to make optimal crosses in developing biofortified barley. GWAS revealed information on the SNP markers associated with Fe and Zn content in barley grains. Their validation in different materials could demonstrate their effectiveness and be applied to the breeding of biofortified barley.

## Figures and Tables

**Table 1:** The population types were distributed into five subpopulations.

Subpopulation	Cultivar	Improved Line	Landrace	Total
1	14	3	74	91
2	4	16	35	55
3	6	3	230	238
4	19	2	16	37
5	52	4	18	74
Total	95	28	373	495

**Table 2:** Annotation of significant SNPs associated with Zn using BARLEX, BarleyVarDB and Barleymap database; for a complete list, see Supplement Table S3.

Trait	SNP	Chr	Pos	*p*	Adjusted *p*	R^2^	Gene ID	Candidate Gene
Zn_2017_18	JHI-Hv50k-2016-198443	3H	598684782	0.000054068	0.002378992	3.426	HORVU3Hr1G082300	Protein NRT1/PTR FAMILY 5.10
Zn 2017_18	JHI-Hv50k-2016-263938	4H	615385811	0.00005644	0.00248336	3.409	HORVU4Hr1G080420	Tetratricopeptide repeat protein 7A
Zn_2017_18	JHI-Hv50k-2016-184521	3H	498961078	0.000066521	0.002926924	3.342	HORVU3Hr1G065530	Zinc finger BED domain-containing protein 1
Zn_2017_18	JHI-Hv50k-2016-184502	3H	498958727	0.000068109	0.002996796	3.332	HORVU3Hr1G065530	Zinc finger BED domain-containing protein 1
Zn_2017_18	JHI-Hv50k-2016-198449	3H	598790707	0.000068898	0.003031512	3.328	HORVU3Hr1G082310	5′-3′ exoribonuclease 3
Zn_2018_19	JHI-Hv50k-2016-232541	4H	26342827	0.000056102	0.001346448	3.419		marker for oxidative stress response protein
Zn_2018_19	SCRI_RS_115755	3H	664905936	0.000056102	0.001968216	3.265	HORVU3Hr1G102520	Nascent polypeptide-associated complex subunit alpha-like protein 3
Zn_2018_19	JHI-Hv50k-2016-44040	1H	518159560	0.00010246	0.00245904	3.175	HORVU1Hr1G077680	Ethylene-responsive transcription factor 1B
Zn_2018_19	JHI-Hv50k-2016-44085	1H	518229092	0.00010246	0.00245904	3.175	HORVU1Hr1G077710	FAR1-related sequence 5
Zn_2018_19	JHI-Hv50k-2016-73691	2H	29669343	0.00012464	0.00299136	3.096	HORVU2Hr1G013690	Undescribed
Zn_2018_19	JHI-Hv50k-2016-73694	2H	29669609	0.00012464	0.00299136	3.096	HORVU2Hr1G013690	Undescribed
Zn_2018_19	JHI-Hv50k-2016-73663	2H	29624393	0.0001632	0.0039168	2.988	HORVU2Hr1G013680	Elongation factor 1-alpha

**Table 3:** Annotation of significant SNPs associated with Fe using BarleyVarDB and Barleymap database; for a complete list, see Supplementary Table S2.

Trait	SNP	Chr	Pos	*p*	Adjusted *p*	R^2^	Gene ID	Candidate Gene
Fe 2018–2019	JHI-Hv50k-2016-114559	2H	690696206	0.000198	0.00396	2.925	HORVU2Hr1G100330	Nuclear pore complex protein-related
Fe 2018–2019	JHI-Hv50k-2016-260401	4H	598066009	0.000222	0.00444	2.88	HORVU4Hr1G075360	Alanine:glyoxylate aminotransferase 2
Fe 2017–2018	JHI-Hv50k-2016-197724	3H	595054022	0.0000312	0.0013104	3.584	HORVU3Hr1G081540	Transcription initiation factor TFIID subunit 9
Fe 2017–2018	JHI-Hv50k-2016-197775	3H	595221701	0.0000869	0.0036498	3.177	HORVU3Hr1G081590	4;5-DOPA dioxygenase extradiol
Fe 2017–2018	JHI-Hv50k-2016-457547	7H	32324334	0.0000978	0.0041076	3.13	HORVU3Hr1G081580	4;5-DOPA dioxygenase extradiol
Fe 2017–2018	JHI-Hv50k-2016-488291	7H	498765300	0.000105	0.00441	3.103	HORVU7Hr1G082900	23S rRNA (uracil(1939)-C(5))-methyltransferase RlmD

## Data Availability

Not applicable.

## Supplementary Materials

The following supporting information can be downloaded at: https://www.mdpi.com/article/10.3390/plants11101349/s1, Figures S1–S5: The frequency distributions with repeated checks, box plots, and descriptive statistics of adjusted means for zinc and iron. Tables S1–S3. Table S1: The list of genotypes used in our study and their corresponding Fe and Zn content values, including descriptive statistics for 2017–2018 and 2018–2019 crop seasons.
