# Supplementary material for: A Genome-Wide Association Study Identifying Single-Nucleotide Polymorphisms for Iron and Zinc Biofortification in a Worldwide Barley Collection
Source: Plants (Basel). 2022 May 19;11(10):1349. doi: 10.3390/plants11101349 (PMC9148054; doi:10.3390/plants11101349)
Supplement: Supplementary file 1 [file plants-11-01349-s001.zip › plants-1651066-Supplement Figures.pdf]

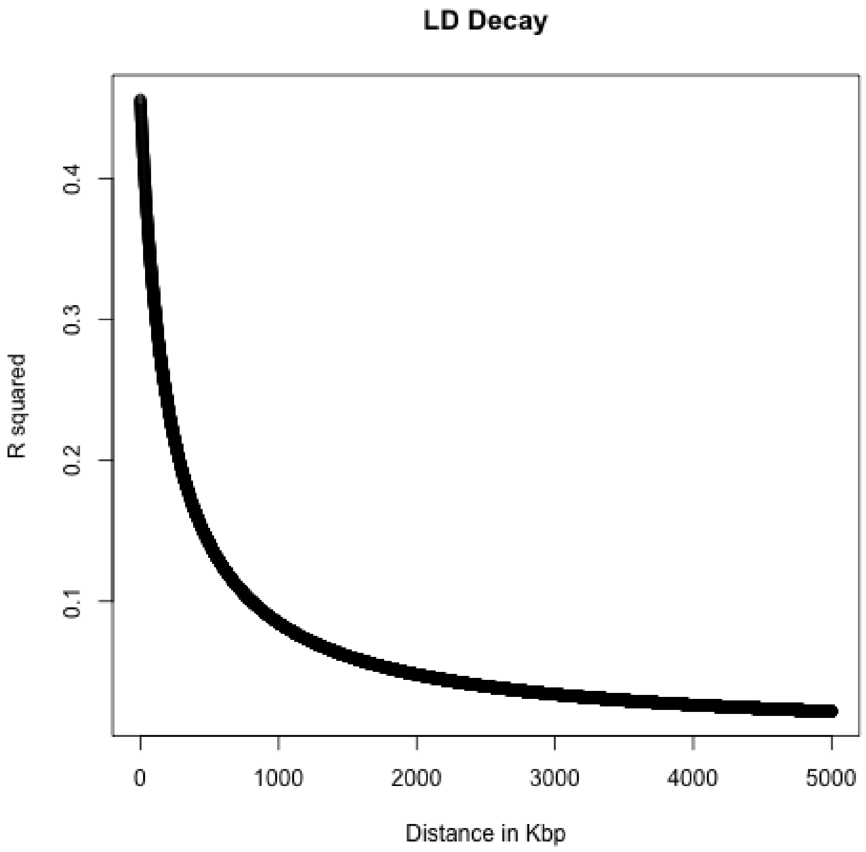

Figure S1. Plot of Linkage disequilibrium LD with r^2 values plotted against physical distances in Kbp.

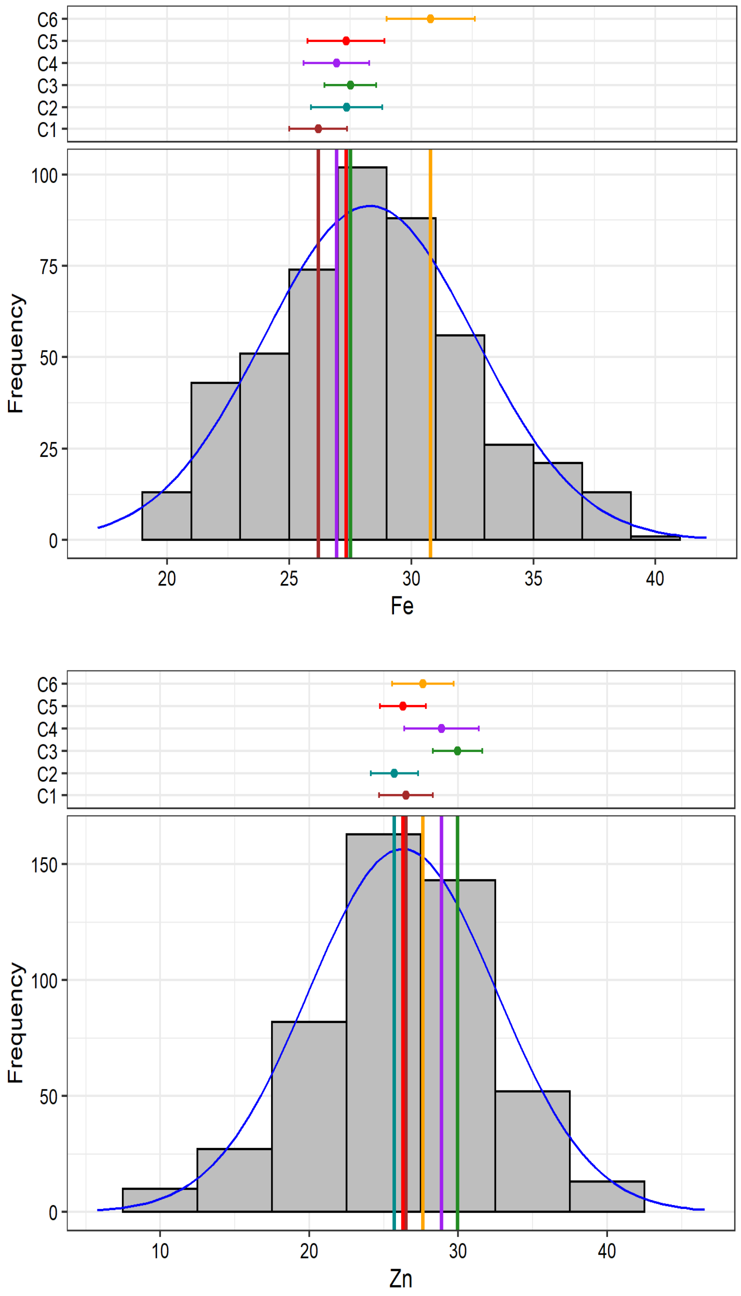

Figure S2. Frequency distribution with checks for Zn and Fe from the 2017-2018 crop season. C1, C2, C3, C4, C5 and C6 are checks (controls) accessions Alanda 01, Rihane, Moralis, Carre 42, Djebali and Marocaine 280, respectively, that are replicated in each blocks, in an Augmented design (see texts for details).

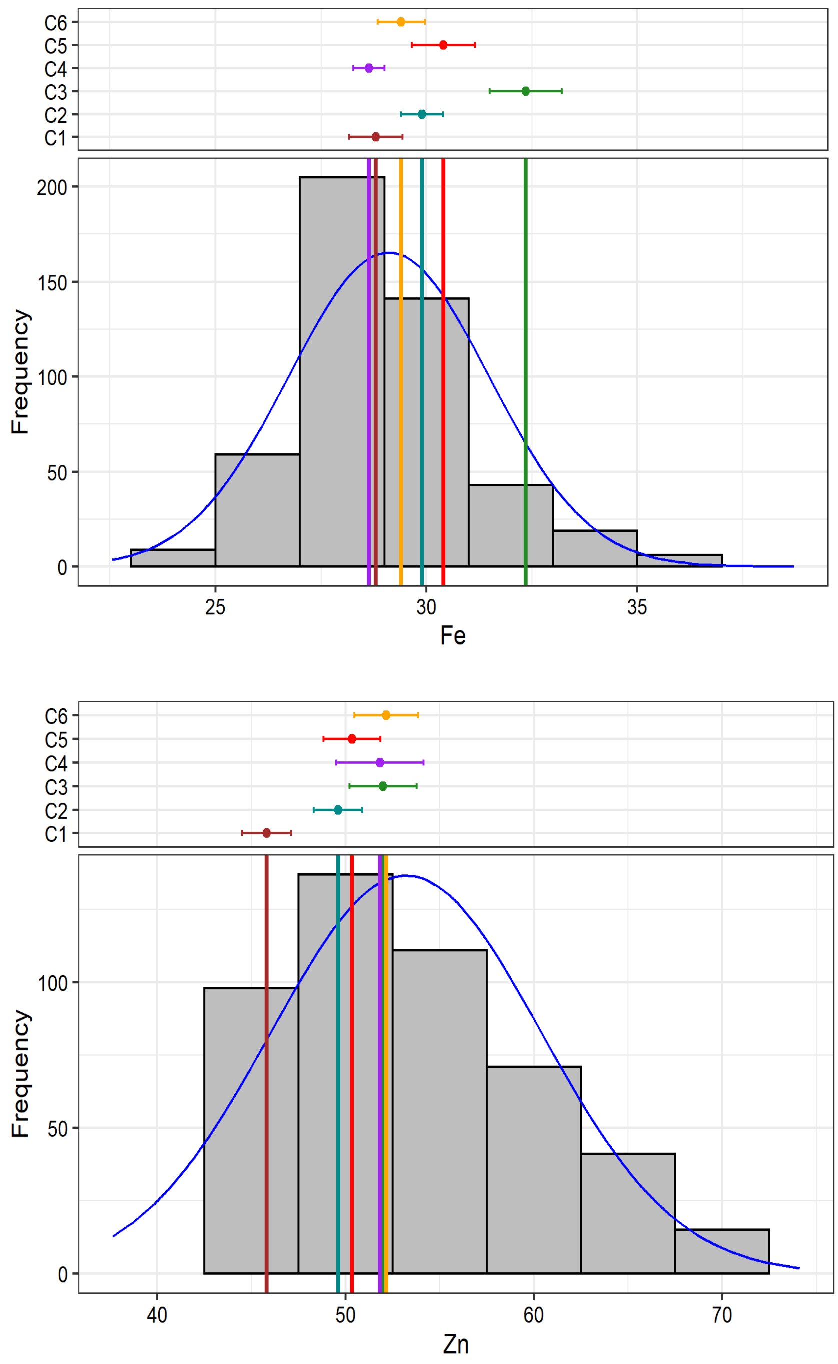

Figure S3. Frequency distribution with checks for Zn and Fe from the 2018-2019 crop season. C1, C2, C3, C4, C5 and C6 are checks (controls) accessions Alanda 01, Rihane, Moralis, Carre 42, Djebali and Marocaine 280, respectively, that are replicated in each blocks, in an Augmented design (see texts for details).

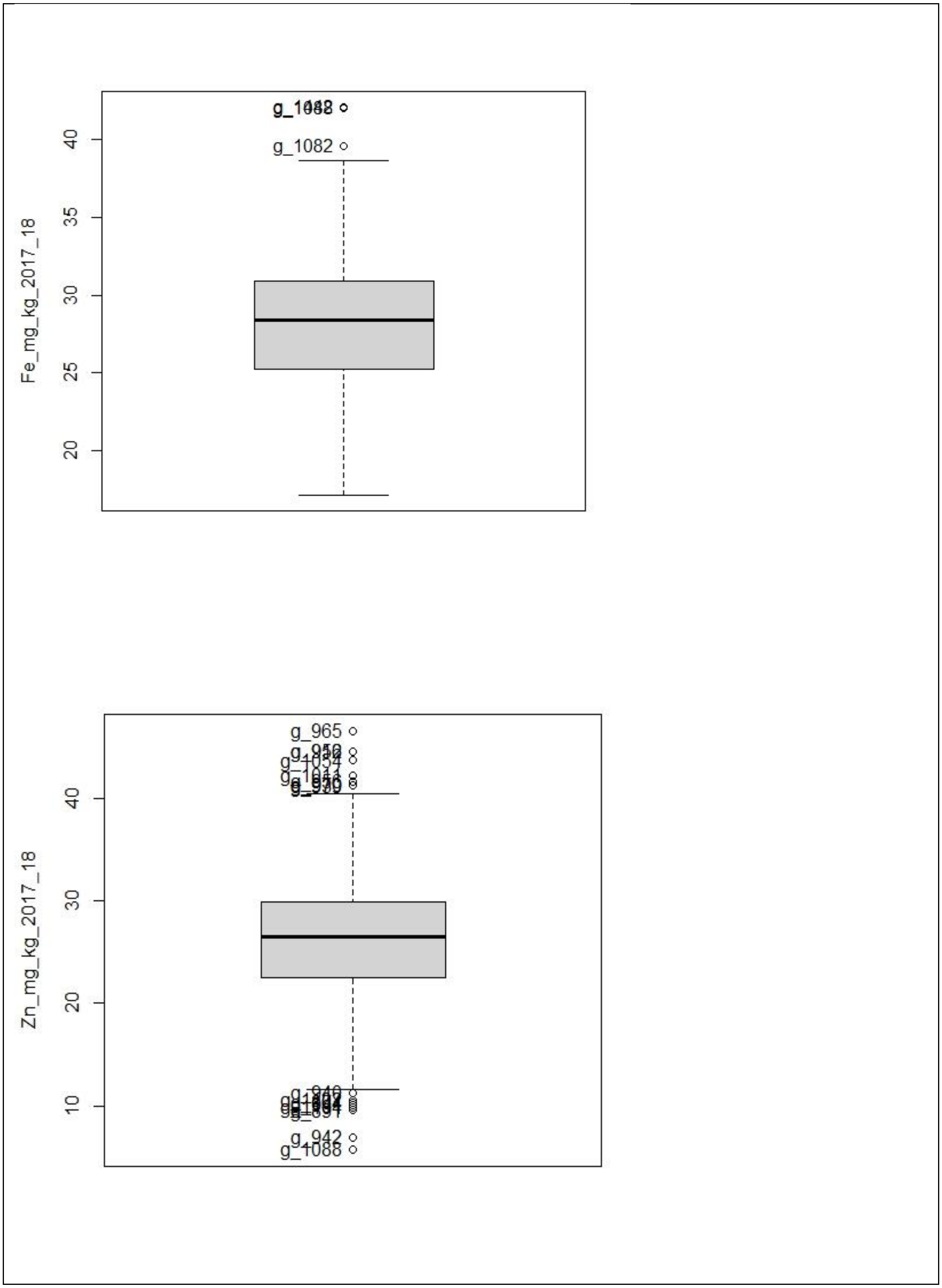

Figure S4. Box plot for Zn and Fe from the 2017-2018 crop season

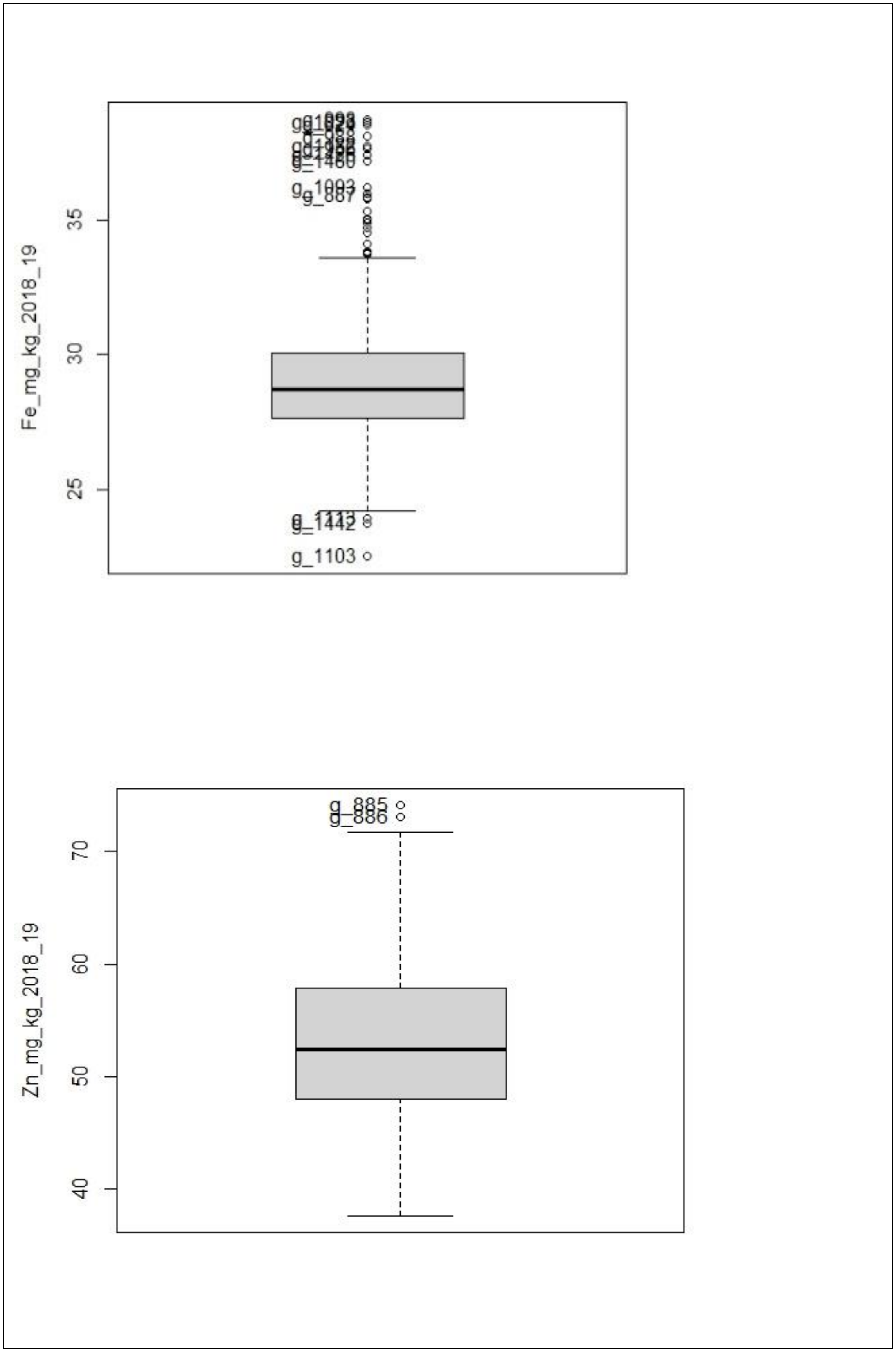

Figure S5. Box plot for Zn and Fe from the 2018-2019 crop season
